# Needle EMG induced muscle bleeding complication after guideline approved discontinuation of anticoagulation

**DOI:** 10.1016/j.cnp.2021.02.005

**Published:** 2021-03-26

**Authors:** Michael Bartl, Arne Krahn, Joachim Riggert, Walter Paulus

**Affiliations:** a Department of Clinical Neurophysiology, University Medical Center, Goettingen, Robert Koch Str. 40, D37075 Göttingen, Germany; b Department of Transfusion Medicine, University Medical Center, Goettingen, Robert Koch Str. 40, D37075 Göttingen, Germany

**Keywords:** Electromyography, Bleeding, Anticoagulation, Non-steroidal anti-inflammatory drugs

## Abstract

**Introduction:**

Needle electromyography (EMG) is an essential part of electrodiagnosis (EDX) in neuromuscular disorders. As in all invasive procedures there is a risk of bleeding complications, but which is rare according to the current literature. Controlled, prospective studies that include patients under anticoagulation or antiplatelet therapy are lacking and would be difficult to conduct.

**Case reports:**

We describe two patients with no history of coagulopathy who developed an intramuscular hematoma after needle EMG. They had been under therapeutic anticoagulation but this had been discontinued, and their standard coagulation parameters had returned to normal prior to the EMG. One patient was found to have a rare genetic defect in thromboxane synthesis with associated markedly impaired platelet aggregation, while no obvious cause of the bleeding was found in the second patient. However, it could have been due to an unexpectedly strong anticoagulatory response to the oral anticoagulant apixaban.

**Conclusion:**

One must be aware of the increased risk of bleeding events in patients with therapeutic anticoagulation. These can occur even when the recommendations regarding discontinuation of anticoagulant drugs for the procedure have been followed. The patient must be actively questioned for ongoing use of NSAIDs, and if pain therapy is required alternatives with no antiplatelet activity should be given. A larger data pool of adverse EMG events would aid in risk assessment and decision making.

## Highlights

• After needle EMG an intramuscular hematoma, requiring surgical evacuation, occurred.
• Anticoagulation was paused according to guidelines; one patient had an undetected genetic disorder of platelet function.
• Substituting apixaban for enoxaparin may have contributed to the complications.

## 1 Introduction

Needle electromyography (EMG) is an essential part of modern electrodiagnosis (EDX). It provides information on acute and chronic denervation, as well as on myotonic or myopathic disorders. As with any other invasive technique there remains a risk of bleeding, however slight. Previous studies have shown an incidence for bleeding events between 0% and 11%, with a much lower risk for a necessary surgical intervention (Baba et al., 2005, Boon et al., 2012, Butler and Dewan, 1984, Farrell et al., 2003, Gechev et al., 2016, Gertken et al., 2013, Rosioreanu et al., 2005, Vaienti et al., 2005). In general, the risk of a significant bleed has to be weighed against the risks inherent in postponing or cancelling the EMG.

The guidelines for EDX have special recommendations for dealing with patients receiving anticoagulation (American Association of Electrodiagnostic Medicine. Guidelines in electrodiagnostic medicine, 1999, Menkes and Pierce, 2019, Stålberg et al., 2019, Tankisi et al., 2020). These include performing coagulation tests, using small diameter needles, and avoiding deep or non-compressible muscles (e.g. paraspinal, psoas) or those which might develop a compartment syndrome, e.g. the tibialis anterior muscle. So far, there are no general recommendations to discontinue anticoagulants or platelet aggregation inhibitors prior to EMG (American Association of Electrodiagnostic Medicine. Guidelines in electrodiagnostic medicine, 1999, Gertken et al., 2013), in particular not with respect to the new class of oral anticoagulants, i.e. the non-vitamin K antagonist oral anticoagulants (NOACs), such as apixaban, dabigatran, rivaroxaban and edoxaban. The American Association of Neuromuscular and Electrodiagnostic Medicine guideline recommends normalizing the coagulation when necessary, only sampling small superficial muscles, and applying local pressure after removing the needle. For surgical procedures with a low risk of bleeding, it is recommended to temporarily discontinue oral anticoagulant therapy (OAT). NOACs are generally discontinued on the evening before the procedure and started again six hours afterwards. For surgical procedures with a high risk of bleeding complications, the NOAC should be paused at least three days in advance, or even earlier when renal function is reduced. Bridging is not deemed necessary with NOAC, but coumarin derivatives should be bridged with heparin (Kelley et al., 2016, Spyropoulos et al., 2016). The literature describes a bleeding risk of up to 2.2% after central venous catheterization with a platelet count >50,000/µL and an international normalized ratio (INR) < 3 (Wolfe and Kress, 2016).

Special care is required when the coagulopathy is due to illness, e.g. thrombopenia, disseminated intravascular coagulopathy, but the literature does not provide any recommendations for this constellation. In view of the possible complications the indication for an EMG must be certain. Furthermore, the duration and scope of the prodecure should be limited to the bare minimum. The recommendations in the literature to avoid EMG in patients with a platelet count lower than 50,000/µL, an INR higher than 1.5–2.0, or a prothrombin time longer than 1.5–2.0 s should be taken into consideration (Gertken et al., 2013).

We describe here two patients with oral anticoagulation for atrial fibrillation in whom serious bleeding complications occurred following a needle EMG. During the five years prior to these events, a total number of 4535 needle EMGs have been performed in the department, including examinations with multiple investigated muscles. No comparable complication has occurred.

## 2 Case report

### 2.1 Patient 1

An 85-year-old man presented with a progressive gait disorder that had developed over two years with numbness, tingling and pain radiating from the right knee into the foot. There was a possible paresis of hip flexion and knee extension. Magnetic Resonance Imaging (MRI) revealed lumbar degeneration with possible compression of the right L4/L5 root. Nerve Conduction Studies (NCS) revealed markedly decreased Compound Muscle Action Potential (CMAP) amplitudes of the right peroneal and tibial nerve with normal sural nerve potentials. Prior to the EMG he has been taking the vitamin K antagonist phenprocoumone. In accordance with our standard operating procedure, phenprocoumone had been discontinued and replaced with subcutaneous enoxaparin 80 mg five days prior to the EMG. The latter was omitted the day before and resumed the day following the EMG. There was no personal or family history of bleeding disorders. The patient had been taking ibuprofen (600 mg PO t.i.d.) for several weeks for his leg pain, and the medication was continued the days before, during and after the performed EMG.

A 37 mm × 26 G concentric bipolar needle electrode was used for the EMG of the left and right anterior tibial muscles, and the right iliopsoas, vastus lateralis, and rectus femoris muscles. No acute denervation potentials were detected.

**Fig. 1:**
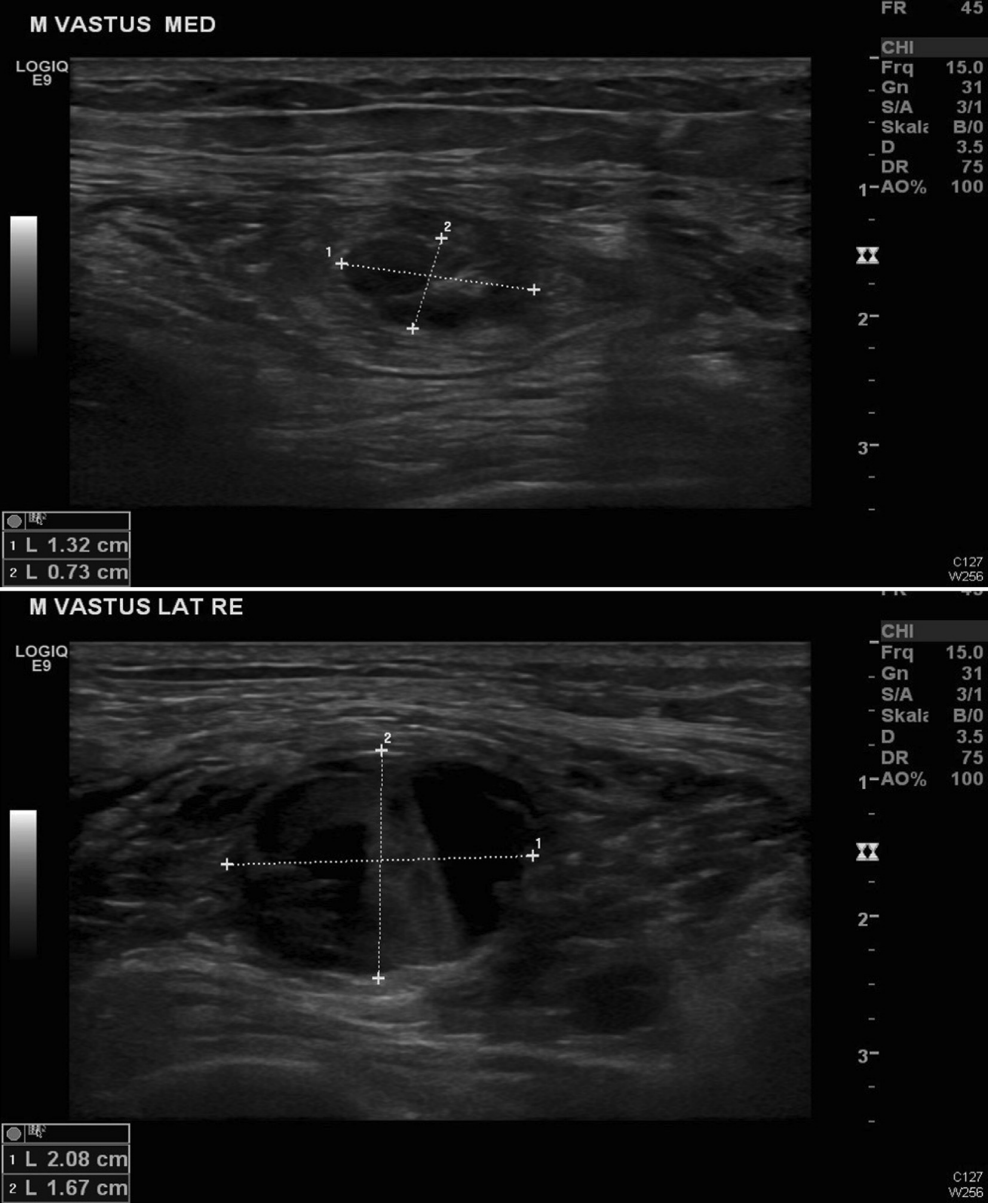
Sonographic evidence of an intramuscular hematoma: a) in the lateral vastus muscle (ca. 2 × 2 × 28 cm), and b) in the medial vastus muscle (ca. 1.5 × 1 × 1.5 cm) (Patient 1).

The right thigh became painfully swollen about 36 h after the EMG. Sonography revealed a large (ca. 2 × 2 × 28 cm) intramuscular hematoma in the vastus lateralis muscle and a smaller one in the medial vastus muscle (ca. 1.5 × 1 × 1.5 cm) in regions corresponding to the EMG needle puncture sites (Fig. 1). Peripheral circulation, sensibility and motor function were preserved. The enoxaparin injections were continued to prevent the development of intracardial thrombi. During the next 24 h the swelling had increased to encompass the entire thigh and had spread to the groin and the knee joint necessitating surgical hematoma evacuation. Six units of blood were required to replace the massive intraoperative blood loss and to correct the postoperative hemoglobin level of 6.0 g/dL.

### 2.2 Patient 2

The second patient was an 80-year-old man who had been hospitalized one week previously because of an exacerbated Chronic Obstructive Pulmonary Disease presented in the emergency department with a suspected complex-focal epileptic seizure. Neurological examination revealed distal paraparesis with grade 4 (Medical Research Council) dorsal extension and eversion of both feet, distal-symmetric sensory deficits, distal areflexia of the legs, and an unsteady gait. He had previously suffered a transient ischemic attack and was under oral anticoagulation with phenprocoumon. There was no family or personal history of bleeding events.

The NCS only showed slightly reduced CMAP amplitudes in both peroneal nerves but there were no pathological changes in the MRI that could explain the clinical features. Bilateral needle EMGs were performed of the tibialis anterior, the gastrocnemicus, and the vastus lateralis muscles after having adapted the anticoagulation regimen according to our internal standard operating procedures (see above). The EMG revealed bilateral acute and chronic denervation potentials. Anticoagulation was started again with oral apixaban 5 mg b.i.d. on the evening of the EMG day. The patient’s weight was 80 kg, his creatinine blood level normal.

**Fig. 2:**
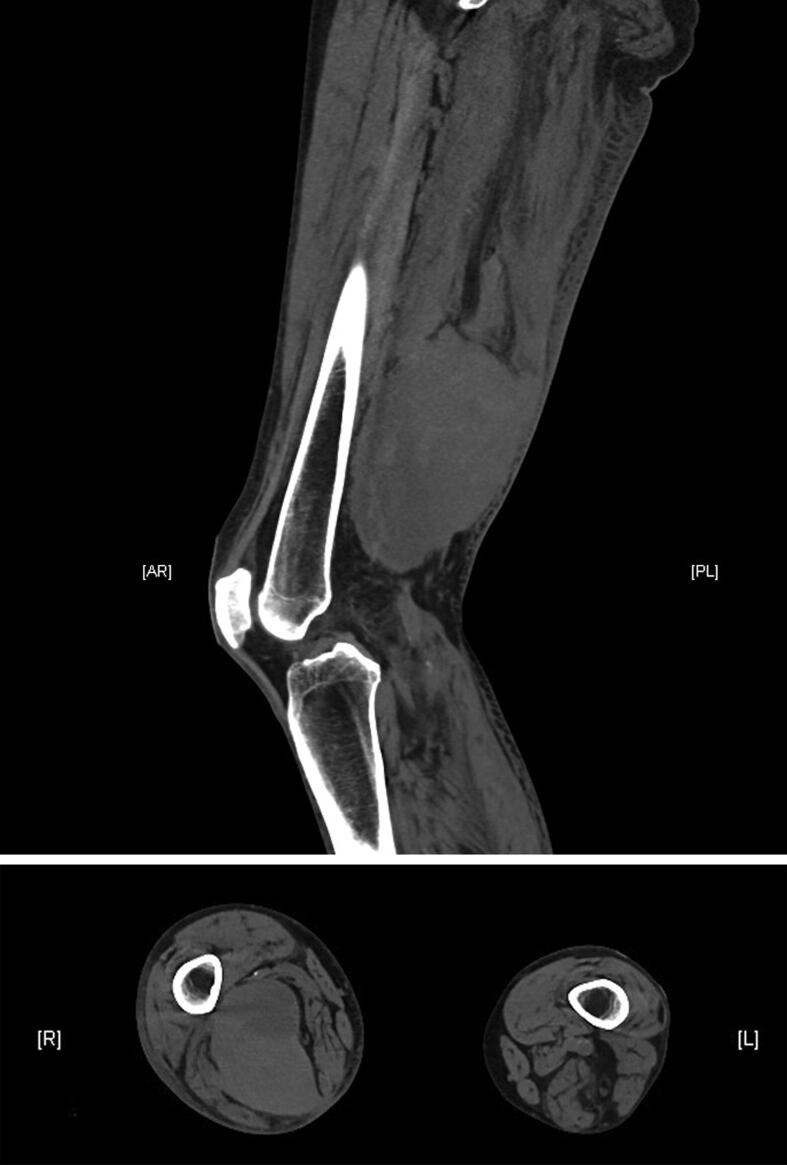
Computed tomography (CT) image of a) a partially intramuscular hematoma located in the dorsal thigh (15 × 10 × 7,5cm) and b) compressing the sciatic nerve (Patient 2).

The day after the EMG the patient complained of swelling, induration and pain in the right dorsal thigh. An approximately 10 × 8 × 5 cm hematoma was found the following day, initially without compressing effect and preserved peripheral circulation, sensibility and motor function. Apixaban was discontinued and enoxaparin s.c. was started. The pain became more severe over the next days, and after four days the patient suddenly developed paralysis of extension and flexion of the right foot. Computer tomography revealed a partially intramuscular hematoma in the dorsal thigh (15 × 10 × 7.5 cm) with compression of the sciatic nerve (Fig. 2). The hematoma was successfully evacuated but the paralysis remained. No further coagulation data are available because the patient did not attend the follow-up examination.

To exclude congenital or acquired disorders of hemostasis and blood clotting, we performed extensive coagulation tests including platelet function tests in both patients (Appendix Table 1, Table 2).

**Table 1:** Coagulation parameters.

	Patient 1					Patient 2			
	EMG	Post-Surgery				EMG			
	pre-EMG	Day 0	Day 1	Day 4	Day 11	pre-EMG day	EMG day	Day 1 post-EMG	Day 6 post-EMG
TPqZ (Quick) (%)	104	92	82	81	90	70	86	83	88
TPZ (INR)	1	1	1.1	1.1	1.1	1.2	1.1	1.1	1.1
Fibrinogen (mg/dl)		335		86	488			244	389
aPTT (sec)	30	31	24	28	34	31		29	27

Patient 1: Surgery was performed nine days after the EMG. Patient 2: Surgery was performed six days after the EMG.

**Table 2:** Platelet function tests.

		Patient 1 without ibuprofen	Patient 1 with ibuprofen	Patient 2 without ibuprofen
Maximal aggregation (%) normal range 50 – 100%	ADP 12,5 µl (50–100)	61	52	88
	ADP25 µl (50–100)	65	58	87
	Kollagen 12,5 µl (50–100)	73	72	89
	Kollagen 25 µl (50–100)	76	83	83
	Arachidonic acid 50 µl (50–100)	4 *	3 *	83
	Ristocetin 50 µl (50–100)	75	81	90
Slope of aggregation (%/min)	ADP 125 µl	36	43	44
	ADP25 µl	33	43	46
	Kollagen 12,5 µl	34	34	46
	Kollagen 25 µl	41	41	47
	Arachidonic acid 50 µl	3	7	34
	Ristocetin 50 µl	45	42	57
In vitro bleeding time (sec)	Kollagen/ADP (62–104)	67	62	69
	Kollagen/Epinephrin (83–170)	85	136	70
Thrombocytes (×10^9 pro µL)		142	175	434

Regarding the first patient we investigated a possible platelet function defect by reexposure to ibuprofen, which was discontinued after the hematoma had been evacuated. The initial platelet function tests were performed several months after the occurrence of the bleeding event with the patient abstaining from taking ibuprofen (Results are shown in the Appendix). With a normal platelet count (142,000/µL) we found values for platelet aggregation after stimulation with adenosine diphosphate, collagen, and ristocetin that were low but still in the normal range. However, after stimulation with arachidonic acid there was virtually no platelet aggregation. These tests were repeated several months later after the patient had been on ibuprofen 600 mg t.i.d. for three days. The results were nearly identical: normal cell counts and *in vitro* bleeding times, normal platelet aggregation after addition of collagen and ristocetin, and no aggregation after stimulation with arachidonic acid. Aggregation under ADP stimulation was slightly reduced.

The fact that in patient 1 the platelets responded to all stimulatory substances except arachidonic acid, which was unable to induce platelet aggregation even in the absence of ibuprofen led to the diagnosis of an aspirin-like defect (details below) (Appendix Table 2) (Weiss and Ames, 1973).

No defect nor any other pathological coagulation test result was found that would explain the bleeding incident in Patient 2 (see Appendix). His management differed only in that his post-EMG anticoagulation was with apixaban instead of enoxaparin (Goldhaber et al., 2011).

## 3 Discussion

Complications of needle electromyography are very rare (<1/10,000) (American Association of Electrodiagnostic Medicine, 1999). Even rarer are bleeding events that require surgical interventions. A recent review (London, 2017) claimed that necessary electrophysiological diagnostic procedures could be performed in patients under antiplatelet medications, or in patients taking warfarin with an INR below 3.0. Furthermore, the patients should not be required to discontinue the therapeutic anticoagulation prior to the procedure. With an INR greater than 3.0, the procedure could be performed by a specialist paying special attention. If the anticoagulation dose was intended to be reduced in any case, it would seem reasonable to wait until the INR was in a normal range (Gertken et al., 2013, London, 2017). The American Association of Neuromuscular and Electrodiagnostic Medicine recommends abstaining from performing an EMG in patients with platelet counts <50,000/µL and an INR > 1.5–2.0 (Gertken et al., 2013). A survey showed that most electromyographers continue to perform needle EMGs in patients who are taking one of the new oral anticoagulation drugs (NOACs) or antiplatelet drugs, and that 90% of them felt comfortable with this (Lee and Kushlaf, 2018). Even if the amount of valid data is limited, clinically relevant bleeding complications in patients taking NOAC seem not to be significantly more frequent than in those using coumarin derivates. Variable practices are followed when performing EMG of the paraspinal and facial muscles. Few patients received a paraspinal EMG, further, no studies of bleeding complications are available for EMG of the facial muscles (Lee and Kushlaf, 2018). Whereas pausing anticoagulation is widely practiced, discontinuing the platelet inhibitors is not usually required. Studies in large cohorts of patients taking ibuprofen during surgical procedures have not shown an increased incidence of bleeding complications (Kelley et al., 2016). The combination of OAT and Ibuprofen may have an increased risk of bleeding complications, even if anticoagulation has been paused (Lamberts et al., 2014). The fact that the platelets responded to all stimulatory substances except arachidonic acid, which was unable to induce platelet aggregation even in the absence of ibuprofen, led to the diagnosis of an aspirin-like defect (Appendix Table 2) (Weiss and Ames, 1973). This is a rare genetic disorder in which an enzyme defect blocks the metabolism of arachidonic acid to thromboxane A2, an important factor in platelet aggregation. This is what is also seen when the activity of cyclooxygenase-1 is blocked by aspirin, hence the name. Most patients with this genetic defect have clinical signs of a coagulation disorder with frequent nose bleeds, easy bruising and prolonged bleeding, but a considerable percentage do not, and they appear normal in standard coagulation screening (Rolf et al., 2009). A bleeding complication was reported in a patient with surgical exploration and cannulation of the femoral vein while on low-molecular weight heparin (LMWH) and acetylsalicylic acid (ASA) without any discontinuation. The LMWH injections were resumed 12 h after surgery, and the patient developed a large hematoma three days later. With a normal heparin anti-factor-Xa level a correlation between the bleeding event and the combination of LMWH and ASA was discussed (Kumar and Solti, 2001). In cardiology an increased awareness for bleedings has been suggested in a survey of patients with anticoagulation and concomitant therapy with an NSAID for pain (Hellfritzsch et al., 2017). When discontinuing the medication for invasive procedures, the risks due to the underlying disease that is the reason for anticoagulation and the benefits and risks of EMG in the respective diagnosis must be weighed critically against each other in each case.

One must be aware of the increased risk of bleeding events in patients with therapeutic anticoagulation, that can occur even when the recommendations regarding discontinuation of anticoagulant drugs have been followed (Lange et al., 2016).

If anticoagulation is continued, it seems justified to follow-up and closely monitor the patients for a longer period because a subacute hemorrhage can occur later than 24 h after the procedure as documented here. A larger pool of reliable data would greatly aid in management decisions.

## 4 Consent for publication

In regard of the case report a consent of publication from the patient was obtained in advance.

## 5 Availability of data and materials

The datasets used and analysed during the current study are available from the corresponding author on reasonable request.

## Declaration of Competing Interest

The authors declare that they have no known competing financial interests or personal relationships that could have appeared to influence the work reported in this paper.
